# Development of machine learning models to predict perioperative blood transfusion in hip surgery

**DOI:** 10.1186/s12911-024-02555-7

**Published:** 2024-06-05

**Authors:** Han Zang, Ai Hu, Xuanqi Xu, He Ren, Li Xu

**Affiliations:** 1 Department of Anesthesiology, Peking Union Medical College Hospital, Chinese Academy of Medical Sciences, Peking Union Medical College, Beijing, 100730 China; 2 Key Laboratory of High Confidence Software Technologies (Peking University), Ministry of Education, Beijing, 100084 China; 3 School of Computer Science, Peking University, Beijing, 100084 China; 4 Beijing HealSci Technology Co., Ltd., Beijing, 100176 China

**Keywords:** Perioperative blood transfusion, Prediction models, Machine learning, Hip surgery, Risk stratification

## Abstract

**Background:**

Allogeneic Blood transfusion is common in hip surgery but is associated with increased morbidity. Accurate prediction of transfusion risk is necessary for minimizing blood product waste and preoperative decision-making. The study aimed to develop machine learning models for predicting perioperative blood transfusion in hip surgery and identify significant risk factors.

**Methods:**

Data of patients undergoing hip surgery between January 2013 and October 2021 in the Peking Union Medical College Hospital were collected to train and test predictive models. The primary outcome was perioperative red blood cell (RBC) transfusion within 72 h of surgery. Fourteen machine learning algorithms were established to predict blood transfusion risk incorporating patient demographic characteristics, preoperative laboratory tests, and surgical information. Discrimination, calibration, and decision curve analysis were used to evaluate machine learning models. SHapley Additive exPlanations (SHAP) was performed to interpret models.

**Results:**

In this study, 2431 hip surgeries were included. The Ridge Classifier performed the best with an AUC = 0.85 (95% CI, 0.81 to 0.88) and a Brier score = 0.21. Patient-related risk factors included lower preoperative hemoglobin, American Society of Anesthesiologists (ASA) Physical Status > 2, anemia, lower preoperative fibrinogen, and lower preoperative albumin. Surgery-related risk factors included longer operation time, total hip arthroplasty, and autotransfusion.

**Conclusions:**

The machine learning model developed in this study achieved high predictive performance using available variables for perioperative blood transfusion in hip surgery. The predictors identified could be helpful for risk stratification, preoperative optimization, and outcomes improvement.

## Background

Hip surgery has been considered as an effective way for patients with hip diseases to relieve pain, improve function and enhance the quality of life. As the geriatric population grows, there has been an increase in the prevalence of degenerative arthritis and hip fracture. The demand for primary total hip arthroplasty is expected to increase to 572,000 procedures by 2030 and the demand for hip revision surgeries is estimated to double by 2026 in the United States [1]. Perioperative blood loss is common in surgical procedures, particularly in orthopedic cases [2]. Previous findings have shown that hip replacement was the most common procedure associated with blood product transfusion [3, 4]. Some recent studies have revealed the correlation between blood product transfusion and adverse outcomes such as postoperative infection, disease transmission, prolonged duration of hospitalization, and increased morbidity [5–11].

Previous transfusion strategies that rely on clinical experience are widely used in hip surgery, which often lead to the overuse of blood products and unnecessary healthcare costs. Optimizing the assessment and management of blood transfusion has become an urgent medical problem. The new guidelines and strategies for perioperative blood transfusion management continue to develop, however, it has remained a challenge for surgeons and anesthesiologists [12, 13]. Although lower preoperative hemoglobin or anemia have been recognized as major predictors, the requirement for transfusion is still not predicted accurately [14–16].

Given the rapid development of artificial intelligence, machine learning has also expanded in medicine, such as clinical prediction [17–21]. Machine learning refers to algorithms that learn to perform tasks from data, explore combinations, and predict outcomes [22]. It has a better performance in handling enormous data with complex and nonlinear relationships than statistical methods [23]. Recently, there are several studies conducted to predict blood transfusion in craniofacial surgery, liver transplantation surgery, and orthopedic surgery by developing predictive models based on machine learning [24–26]. The above studies generally agree that machine learning algorithms have advantages in predicting the risk of blood transfusion.

To date, few studies have existed that evaluate the application of machine learning prediction models in hip surgery. Our study aimed to develop machine learning models and identify risk factors for perioperative blood transfusion in hip surgery.

## Methods

### Patients

**Fig. 1:**
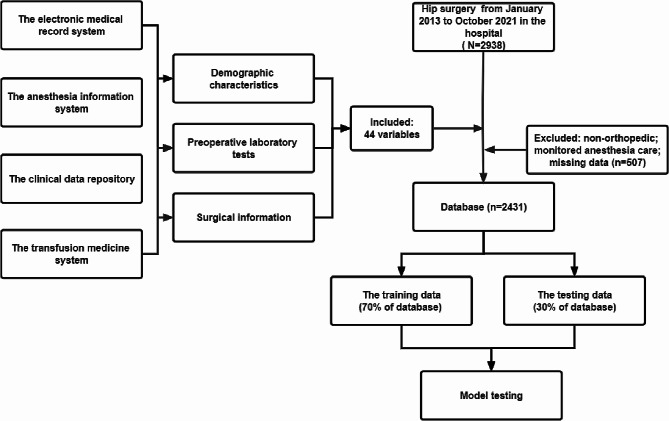
The flowchart of the study

This retrospective cohort study has been approved by the Institutional Review Board of the Peking Union Medical College Hospital (Ethics Approval Number: S-K1757) and informed consent was waived because of retrospective analysis. The study was conducted following the Transparent Reporting of a multivariable prediction model for Individual Prognosis Or Diagnosis (TRIPOD) [27]. The subjects were patients undergoing hip surgery, from January 2013 to October 2021 in the Peking Union Medical College Hospital. In total, 2431 hip surgeries were included in this study. The flowchart with inclusion and exclusion details was displayed (Fig. 1).

### Data collection

All variables were selected based on previous studies, clinical experience, and data availability through system extraction and manual collection. Data were sourced from the electronic medical record system, the anesthesia information system, the clinical data repository, and the transfusion medicine system including demographic characteristics, preoperative laboratory tests, and surgical information (Table 1). Patient demographic characteristics included age, body mass index (BMI), sex, ASA Physical Status, hypertension, diabetes, coronary heart disease, anemia, and medications. ASA Physical Status was assessed by an anesthesiologist before surgery. Anemia was defined for men and women: preoperative hemoglobin < 120 and < 110 g/L, respectively. Medications included anticoagulant or antiplatelet history defined as receiving heparin, warfarin, factor Xa inhibitor, aspirin, or platelet P2Y12 receptor blocker within one week before surgery. Preoperative laboratory tests represented the most recent values before surgery including preoperative hemoglobin, platelet, activated partial thromboplastin time (APTT), prothrombin time (PT), D-dimer, fibrinogen, alanine aminotransferase (ALT), total bilirubin, direct bilirubin, albumin, creatinine, and urea. Surgical information included anesthesia approach, diagnosis, emergency or elective surgery, surgery type, autotransfusion, tranexamic acid use, and operation time. Autotransfusion represented intraoperative cell salvage. Osteoarthritis included primary osteoarthritis and secondary osteoarthritis. The primary outcome was perioperative RBC transfusion that referred to allogeneic RBC transfusion intraoperatively or within 72 h postoperatively. In accordance with the restrictive strategy recommended in guidelines, the transfusion threshold in our institution are: (1) hemoglobin concentration < 80 g/L; (2) hemoglobin concentration < 100 g/L for those with preexisting cardiovascular disease or obvious clinical symptoms [28].

**Table 1:** Patient characteristics of all the data

Variables	Hip surgery (*n* = 2431)
Demographic characteristics	
Age (years)	57 (43, 67)
BMI (kg/m^2^)	24.0 (21.5, 26.6)
Sex (female, %)	1376 (56.6)
ASA Physical status > 2 (%)	372 (15.3)
Hypertension (%)	765 (31.5)
Diabetes (%)	242 (10.0)
Coronary heart disease (%)	145 (6.0)
Anemia (%)	345 (14.2)
Anticoagulant history (%)	389 (16.0)
Antiplatelet history (%)	30 (1.2)
Surgical information	
General anesthesia (%)	2246 (92.4)
Femoral head necrosis (%)	1040 (42.8)
Hip fracture (%)	394 (16.2)
Developmental dysplasia of the hip (%)	303 (12.5)
Osteoarthritis (%)	929 (38.2)
Rheumatoid arthritis (%)	109 (4.5)
Hemophilic arthritis (%)	36 (1.5)
Ankylosing spondylitis (%)	125 (5.1)
Bone tumor (%)	59 (2.4)
Osteoporosis (%)	636 (26.2)
Hip stiffness (%)	45 (1.9)
Systemic lupus erythematosus (%)	170 (7.0)
Sjogren syndrome (%)	36 (1.5)
Emergency surgery (%)	128 (5.3)
Total hip arthroplasty (%)	2010 (82.7)
Artificial femoral head replacement (%)	209 (8.6)
Revision surgery (%)	71 (2.9)
Debridement (%)	35 (1.4)
Lesion excision (%)	39 (1.6)
Autotransfusion (%)	386 (15.9)
Tranexamic acid use (%)	2082 (85.6)
Operation time (min)	112.0 (86.0, 168.0)
Preoperative laboratory tests	
Hemoglobin (g/L)	131(119, 143)
Platelet (10^9^/L)	223 (183, 266)
APTT (s)	27.5 (25.5, 30.1)
PT (s)	11.8 (11.3, 12.3)
D-dimer (mg/L FEU)	0.5 (0.3, 1.3)
Fibrinogen (g/L)	3.1 (2.6, 3.8)
ALT (U/L)	17 (12, 25)
Total bilirubin (µmol/L)	10.0 (7.7, 13.3)
Direct bilirubin (µmol/L)	3.1 (2.4, 4.1)
Albumin (g/L)	40 (37, 43)
Creatinine (µmol/L)	64 (55, 76)
Urea (mmol/L)	5.1 (4.2, 6.3)

Continuous variables were shown by median (interquartile range) and categorical variables were shown by frequency (percentage). Abbreviations: BMI, body mass index; ASA, American Society of Anesthesiologists; APTT, activated partial thromboplastin time; PT, prothrombin time; ALT, alanine aminotransferase; min, minutes

### Data preprocessing

Patients with missing data were eliminated. Missing data were defined as any unknown details for demographic characteristics, preoperative laboratory tests, surgical information, or perioperative blood transfusion. Standardization was performed using the StandardScaler in the continuous variables. Categorical variables were converted to 0 and 1 as input for machine learning models.

### Model training

The first 70% of the data were divided for model training and the latter 30% were divided for model testing based on the time of surgery. The training set was used to construct models, and the testing set was used to evaluate model performances. A univariate analysis was performed in the training set. Only those positive variables that were with a *P* value < 0.05 were considered in the prediction models. Fourteen machine learning models were developed using preoperative variables to predict perioperative blood transfusion, including logistic regression, Ridge Classifier, Random Forest Classifier, Gradient Boosting Classifier, CatBoost Classifier, Ada Boost Classifier, Naive Bayes, SVM-Linear Kernel, Extra Trees Classifier, Light Gradient Boosting Machine, Linear Discriminant Analysis, K Neighbors Classifier, Extreme Gradient Boosting, and Decision Tree Classifier.

For the logistic regression analysis, the step-forward selection was used to identify the most important variables for predicting the outcome. The goal of step-forward selection is to iteratively add variables to the model, starting from an empty model, based on their performances in improving the model’s fit. For machine learning models, all models were developed with hyperparameter tuning through tenfold cross-validation on the training set to optimize performances.

### Model evaluation and explanation

After training in the training set, all the models were evaluated in the testing set. Model performance was compared using metrics of discrimination and calibration. Discrimination was assessed by the area under the receiver operating characteristic curve (AUC). Additionally, the accuracy, recall, precision, and F1 score of models were also assessed. Calibration was measured by the Brier score [29]. The best-performing machine learning model was decided based on the combination of the highest AUC and the lowest Brier score. Decision curve analysis was developed to evaluate the clinical utility of the best-performing model by calculating the net benefit at different threshold probabilities [30].

We used the SHAP values to perform global variables importance analysis, which has been widely used for machine learning model interpretation [31]. The interpretation was based on the SHAP value of each variable, indicating the impact of variables on the prediction. The SHAP summary plot provided the associations between variables and model predictions. We could visualize the relative contribution of each variable and understand how they affected the model output. The contribution of variables was quantified by SHAP values and displayed from high to low values. A positive SHAP value was associated with higher risk of transfusion and a negative one was related to decreased risk of transfusion.

### Statistical analysis

All analyses were performed through Python 3.7 with sklearn, pycaret, statsmodels, numpy, pandas, seaborn, matplotlib, and shap packages. Continuous variables were described as median with interquartile range and compared by the Mann-Whitney U test. Categorical variables were represented as frequency with percentage and compared by the chi-square test. A value of *P* < 0.05 was considered significant.

## Results

### Patient characteristics

Overall, a total of 2431 hip surgeries were enrolled for analysis (Table 1). 614 (25.3%) hip surgeries received perioperative blood transfusion, including 303 (12.5%) hip surgeries received intraoperative blood transfusion, and 224 (9.2%) hip surgeries received blood transfusion within 72 h after surgery. 87 (3.6%) hip surgeries received intraoperative and postoperative blood transfusion within 72 h after surgery. The average intraoperative blood loss was 1044.7 ± 705.1 ml. All data was divided into training (*n* = 1701) and test (*n* = 730) sets. Perioperative blood transfusion was observed in 458 (26.9%) hip surgeries of the training set and 156 (21.4%) hip surgeries of the testing set. The average intraoperative blood loss was 1114.1 ± 737.6 ml in the training set and 882.9 ± 592.5 ml in the testing set.

### Univariate analysis

In the univariate analysis of the training set, the transfusion group and non-transfusion group differed in BMI, ASA Physical Status > 2, anemia, femoral head necrosis, developmental dysplasia of the hip, osteoarthritis, rheumatoid arthritis, hemophilic arthritis, ankylosing spondylitis, osteoporosis, hip stiffness, total hip arthroplasty, artificial femoral head replacement, revision surgery, debridement, autotransfusion, operation time, hemoglobin, PT, APTT, D-dimer, fibrinogen, ALT, total bilirubin, albumin and creatinine (Table 2).

**Table 2:** Univariate analysis of variables in the training set

Variables	Transfusion (*n* = 458)	No-transfusion (*n* = 1243)	*P* value
Demographic characteristics			
Age (years)	58 (43, 69)	57 (44, 67)	0.631
BMI (kg/m^2^)	23.7 (20.8, 26.5)	24.2 (21.7, 26.7)	0.009*
Sex (female, %)	274 (59.8)	681 (54.8)	0.063
ASA Physical status > 2 (%)	100 (21.8)	147 (11.8)	< 0.001*
Hypertension (%)	150 (32.8)	400 (32.2)	0.823
Diabetes (%)	50 (10.9)	123 (9.9)	0.536
Coronary heart disease (%)	34 (7.4)	69 (5.6)	0.151
Anemia (%)	123 (26.9)	109 (8.8)	< 0.001*
Anticoagulant history (%)	85 (18.6)	191 (15.4)	0.113
Antiplatelet history (%)	7 (1.5)	14 (1.1)	0.505
Surgical information			
General anesthesia (%)	419 (91.5)	1146 (92.2)	0.631
Femoral head necrosis (%)	137 (29.9)	551 (44.3)	< 0.001*
Hip fracture (%)	85 (18.6)	196 (15.8)	0.169
Developmental dysplasia of the hip (%)	48 (10.5)	176 (14.2)	0.047*
Osteoarthritis (%)	124 (27.1)	513 (41.3)	< 0.001*
Rheumatoid arthritis (%)	31 (6.8)	48 (3.9)	0.012*
Hemophilic arthritis (%)	13 (2.8)	17 (1.4)	0.041*
Ankylosing spondylitis (%)	43 (9.4)	45 (3.6)	< 0.001*
Bone tumor (%)	12 (2.6)	33 (2.7)	0.968
Osteoporosis (%)	136 (29.7)	285 (22.9)	< 0.004*
Hip stiffness (%)	17 (3.7)	11 (0.9)	< 0.001*
Sjogren syndrome (%)	9 (2.0)	16 (1.3)	0.303
Systemic lupus erythematosus (%)	25 (5.5)	80 (6.4)	0.457
Emergency surgery (%)	25 (5.5)	54 (4.3)	0.333
Total hip arthroplasty (%)	338 (73.8)	1057 (85.0)	< 0.001*
Artificial femoral head replacement (%)	50 (10.9)	97 (7.8)	0.043*
Revision surgery (%)	27 (5.9)	27 (2.2)	< 0.001*
Debridement (%)	19 (4.2)	12 (1.0)	< 0.001*
Lesion excision (%)	10 (2.2)	23 (1.9)	0.659
Autotransfusion (%)	135 (29.5)	158 (12.7)	< 0.001*
Tranexamic acid use (%)	402 (87.8)	1073 (86.3)	0.435
Operation time (min)	174.8 (111.7, 234.7)	105.5 (84.0, 141.4)	< 0.001*
Preoperative laboratory tests			
Hemoglobin (g/L)	123 (110, 135)	134 (124, 145)	< 0.001*
Platelet (10^9^/L)	216.5 (173, 271)	224 (186.5, 267.5)	0.080
APTT (s)	28.7 (26.0, 32.6)	27.7 (25.6, 30.5)	< 0.001*
PT (s)	12.0 (11.4, 12.6)	11.8 (11.3, 12.3)	< 0.001*
D-dimer (mg/L FEU)	0.8 (0.4, 2.0)	0.5 (0.3, 1.1)	< 0.001*
Fibrinogen (g/L)	3.3 (2.7, 4.0)	3.1 (2.6, 3.7)	< 0.001*
ALT (U/L)	16 (12, 24)	17 (12, 26)	0.013*
Total bilirubin (µmol/L)	9.3 (7.3, 12.7)	10.3 (7.9, 13.5)	< 0.001*
Direct bilirubin (µmol/L)	3.1 (2.4, 4.3)	3.2 (2.4, 4.2)	0.635
Albumin (g/L)	39 (36, 42)	41 (38, 43)	< 0.001*
Creatinine (µmol/L)	64 (54, 75)	65 (56, 76)	0.022*
Urea (mmol/L)	5.1 (4.2, 6.5)	5.0 (4.2, 6.2)	0.131

*Variables with a *P* value < 0.05 were included in the predictive models. Continuous variables were shown by median (interquartile range) and categorical variables were shown by frequency (percentage). Abbreviations: BMI, body mass index; ASA, American Society of Anesthesiologists; APTT, activated partial thromboplastin time; PT, prothrombin time; ALT, alanine aminotransferase; min, minutes

### Multivariate logistic regression analysis

Multivariate logistic regression analysis demonstrated that the following variables were independent risk factors for perioperative blood transfusion: ASA Physical Status > 2 (OR, 1.91; 95%CI, 1.32 to 2.77), autotransfusion (OR, 1.45; 95%CI, 1.02 to 2.06) and longer operation time (OR, 1.02; 95%CI, 1.01 to 1.02) (Table 3). Femoral head necrosis (OR, 0.55; 95%CI, 0.41 to 0.74), osteoarthritis (OR, 0.59; 95%CI, 0.43 to 0.80), and higher preoperative hemoglobin (OR, 0.95; 95%CI, 0.94 to 0.96) were associated with decreased transfusion risk (Table 3).

**Table 3:** Multivariable logistic regression analysis of variables in the training set

Variables	OR (95% CI)	*P* value
ASA Physical status > 2	1.91 (1.32–2.77)	0.001
Anemia	1.02 (0.65–1.62)	0.918
Femoral head necrosis	0.55 (0.41–0.74)	< 0.001
Osteoarthritis	0.59 (0.43–0.80)	0.001
Ankylosing spondylitis	1.40 (0.78–2.53)	0.261
Total hip arthroplasty	1.37 (0.92–2.03)	0.123
Debridement	1.40 (0.57–3.43)	0.460
Autotransfusion	1.45 (1.02–2.06)	0.036
Operation time	1.02 (1.01–1.02)	< 0.001
Hemoglobin	0.95 (0.94–0.96)	< 0.001
PT	1.06 (0.94–1.19)	0.361
D-dimer	1.01 (0.99–1.03)	0.376
Fibrinogen	0.91 (0.79–1.06)	0.226
Albumin	0.98 (0.94–1.02)	0.259

Abbreviations: OR, odds ratio; CI, confidence interval; ASA, American Society of Anesthesiologists; PT, prothrombin time

### Performance of machine learning models

**Fig. 2:**
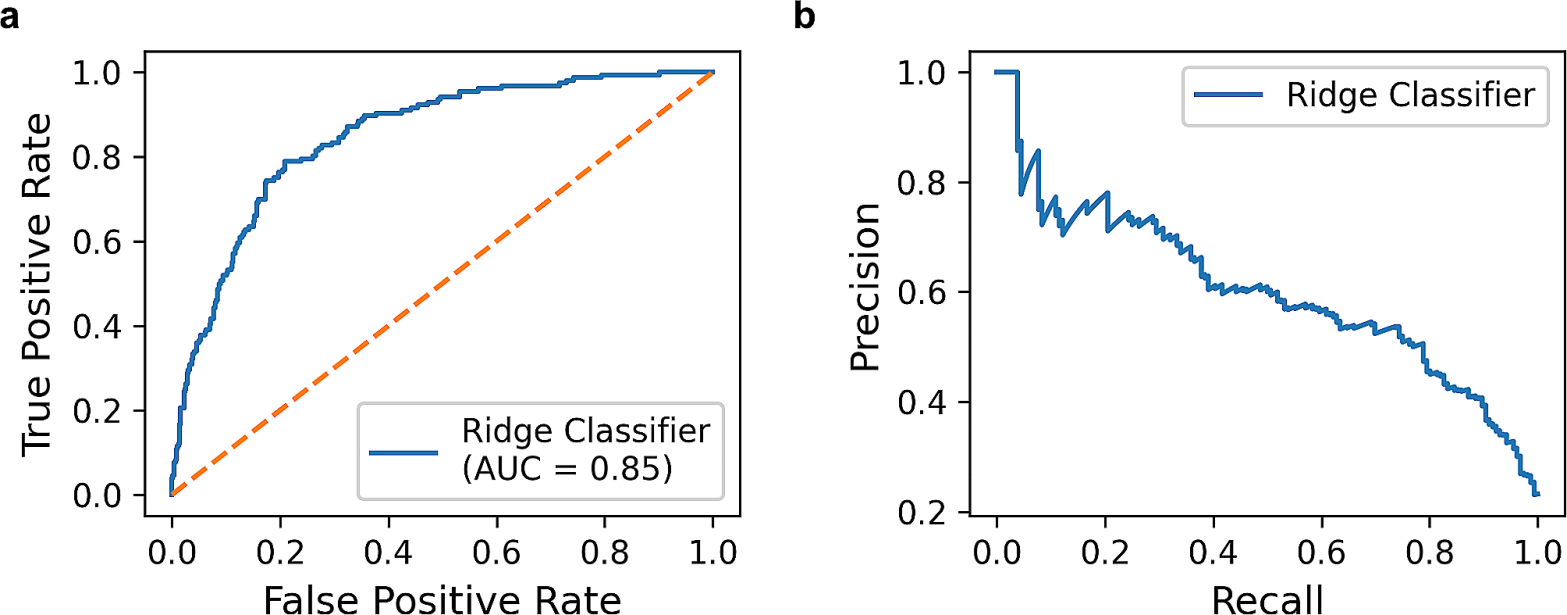
**a** The receiver operating characteristic curve of the Ridge Classifier in the testing set. **b** The precision-recall curve of the Ridge Classifier in the testing set

**Fig. 3:**
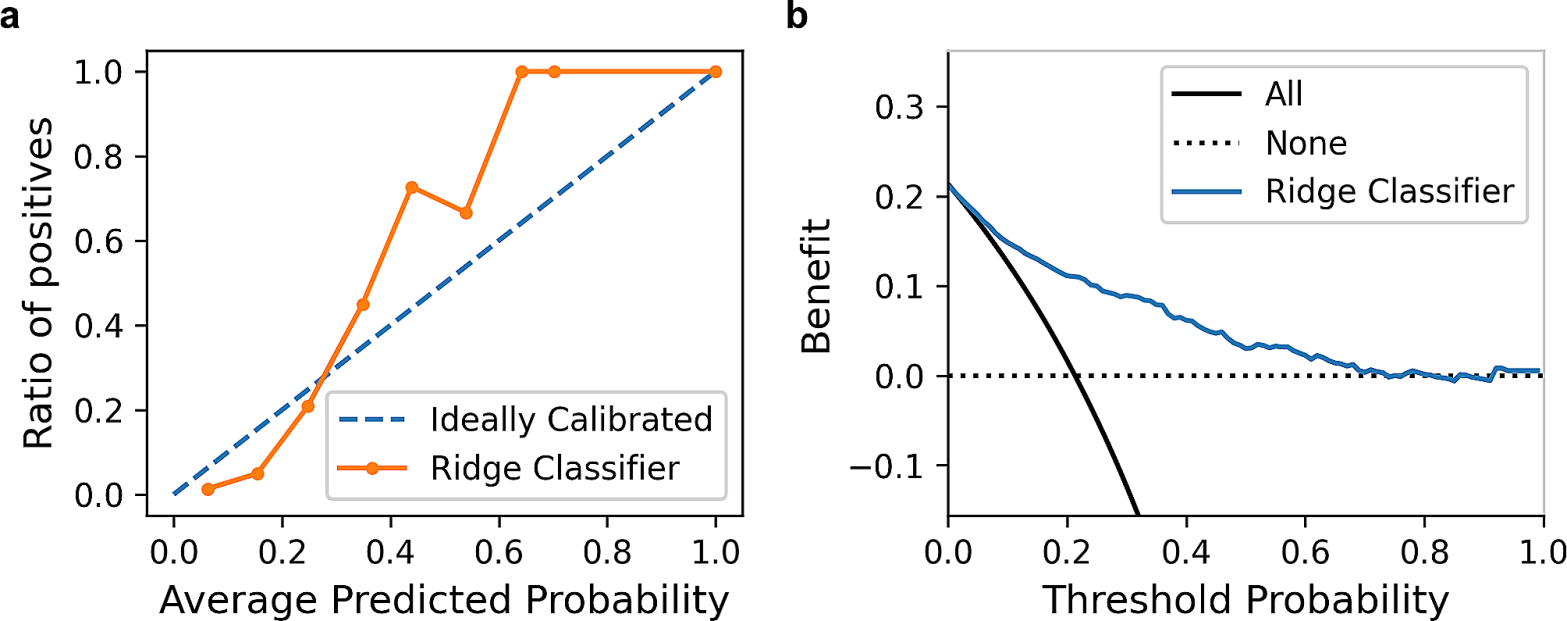
**a** The calibration curve of the Ridge Classifier in the testing set. **b** The decision curve analysis of the Ridge Classifier in the testing set

The testing set of 730 hip surgeries was used to evaluate the predictive abilities of machine learning models. The Ridge Classifier demonstrated the best performance with the highest AUC of 0.85 (95% CI, 0.81 to 0.88) and the lowest Brier score of 0.21. The receiver operating characteristic curve, the precision-recall curve, and the calibration curve of the Ridge Classifier in the testing set were displayed in Fig. 2a and b, and Fig. 3a. The comparison of accuracy, recall, precision, F1 score, and Brier score among all models was also shown in Table 4. The decision curve analysis in the testing set suggested the Ridge Classifier achieved good net benefit for the prediction of perioperative blood transfusion (Fig. 3b).

**Table 4:** Discrimination and calibration metrics of machine learning models in the testing set

Models	AUC (95%CI)	Accuracy	Recall	Precision	F1	Brier
Ridge Classifier	0.85 (0.81–0.88)	0.79	0.78	0.50	0.61	0.21
Extra Trees Classifier	0.85 (0.81–0.87)	0.75	0.87	0.45	0.59	0.25
Ada Boost Classifier	0.85 (0.81–0.87)	0.75	0.81	0.45	0.58	0.25
Linear Discriminant Analysis	0.84 (0.80–0.87)	0.80	0.78	0.51	0.62	0.20
Logistic Regression	0.84 (0.80–0.88)	0.78	0.79	0.48	0.60	0.22
Random Forest Classifier	0.84 (0.80–0.86)	0.75	0.81	0.45	0.58	0.25
CatBoost Classifier	0.82 (0.78–0.86)	0.82	0.67	0.56	0.61	0.18
SVM - Linear Kernel	0.82 (0.78–0.87)	0.78	0.79	0.50	0.61	0.22
Light Gradient Boosting Machine	0.82 (0.78–0.85)	0.71	0.82	0.41	0.55	0.29
Extreme Gradient Boosting	0.81 (0.77–0.84)	0.77	0.69	0.48	0.56	0.23
Gradient Boosting Classifier	0.80 (0.75–0.83)	0.77	0.70	0.47	0.56	0.23
Naive Bayes	0.79 (0.72–0.83)	0.74	0.71	0.43	0.54	0.26
Decision Tree Classifier	0.71 (0.66–0.75)	0.78	0.29	0.46	0.36	0.22
K Neighbors Classifier	0.71 (0.62–0.76)	0.75	0.38	0.42	0.40	0.25

**Fig. 4:**
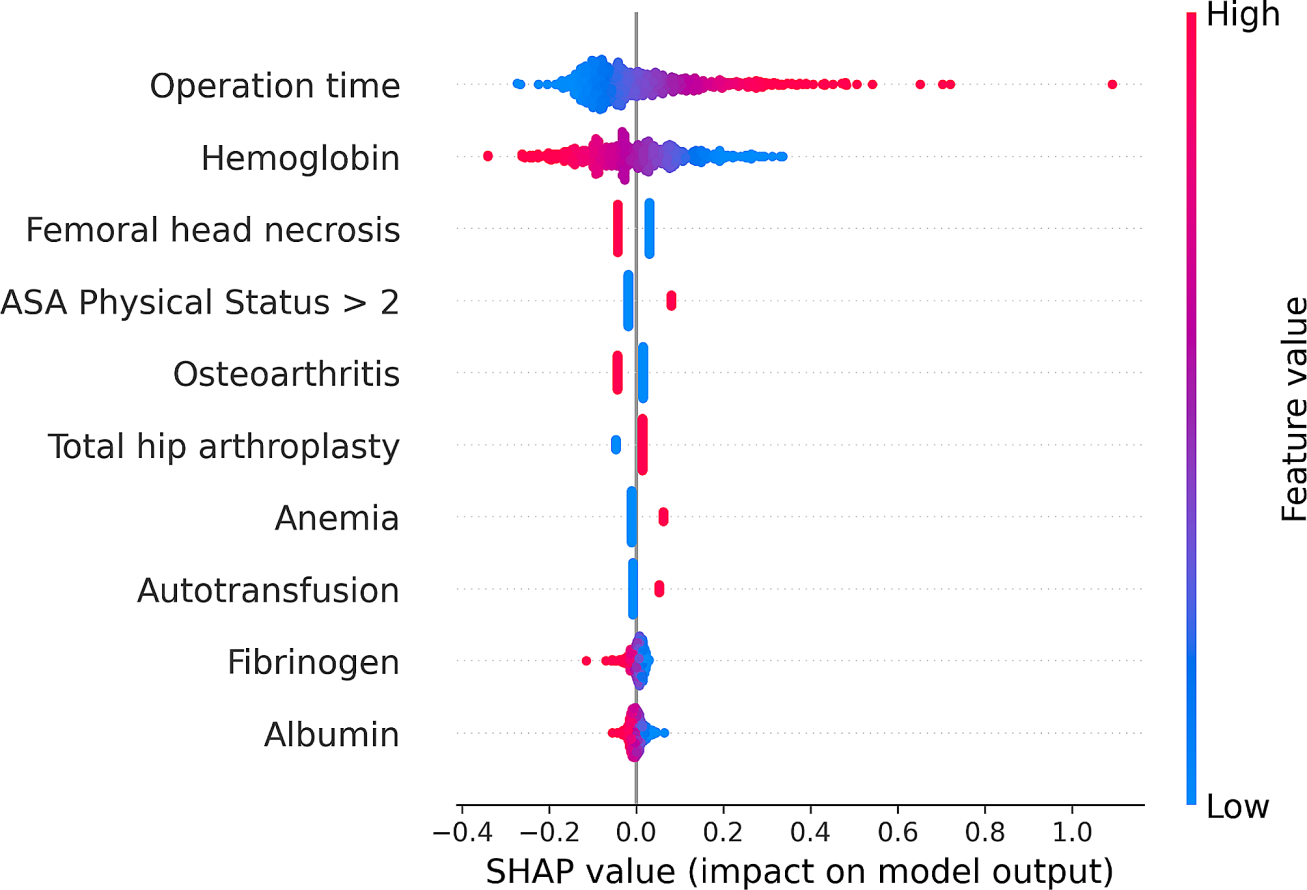
Summary plot for the importance analysis of the top 10 variables in the Ridge Classifier. Each variable was made up of individual dots, each of which was the SHAP value of a sample. Variables with wide distribution indicated strong contribution to model predictions. For continuous variables, red color represented high values and blue represented low values of variables. For categorical variables, the red color represented the presence of variables and the blue color represented the absence of variables. A positive SHAP value (SHAP value greater than 0) was associated with increased transfusion risk and a negative one (SHAP value less than 0) was related to decreased transfusion risk. Abbreviations: ASA, American Society of Anesthesiologists

**Fig. 5:**
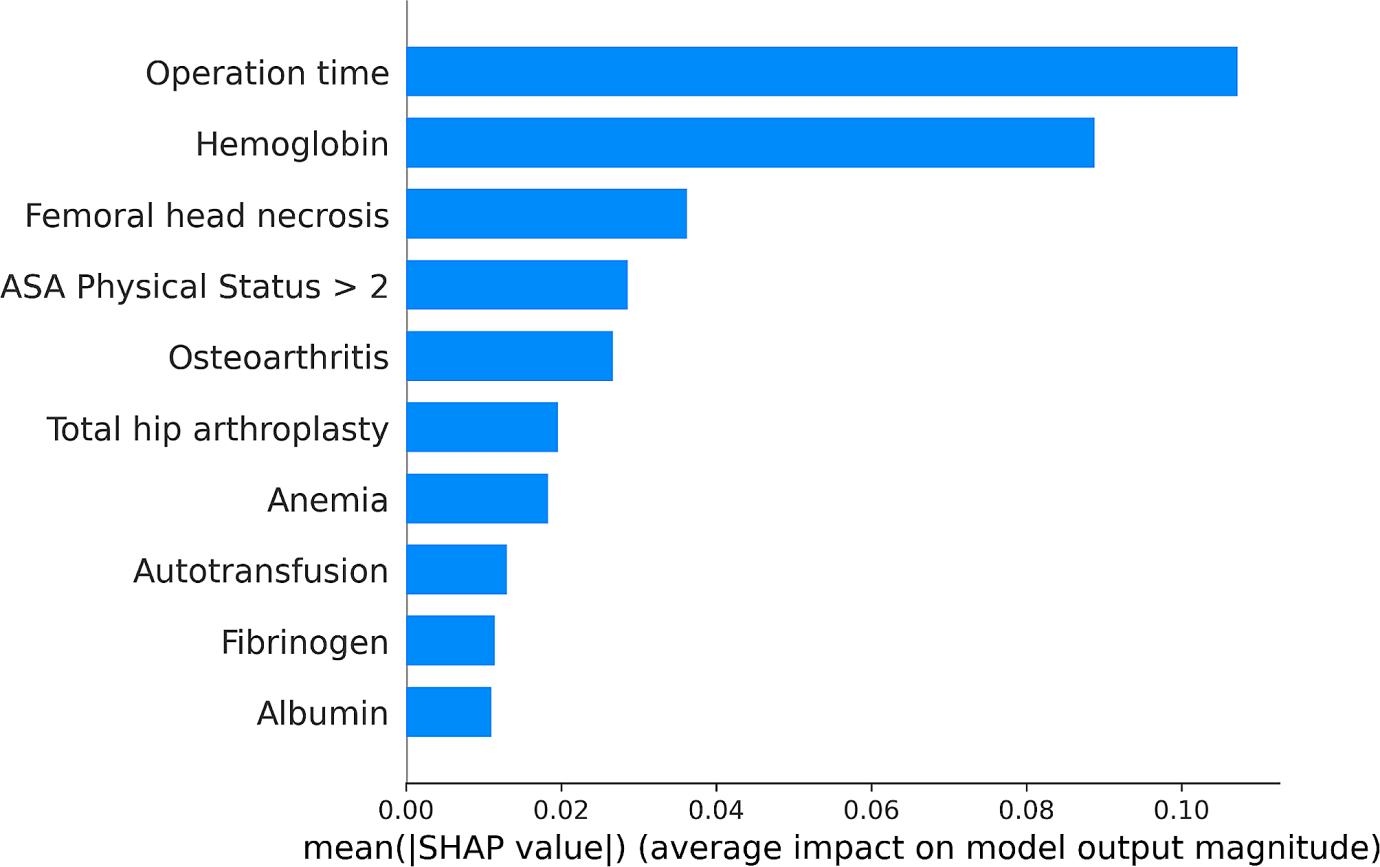
Summary plot for global average impact of the top 10 variables in the Ridge Classifier. It was shown using the average absolute value of all SHAP values of each variable. Abbreviations: ASA, American Society of Anesthesiologists

Further model interpretation was implemented using the SHAP values for the Ridge Classifier. For the global variable importance analysis, the SHAP summary plot showed the top 10 most relevant variables (Figs. 4 and 5). Operation time and preoperative hemoglobin had the greatest average effect on the model prediction. Femoral head necrosis, ASA Physical Status > 2, osteoarthritis, total hip arthroplasty, anemia, autotransfusion, preoperative fibrinogen, and preoperative albumin had the lower average effect. We found that patients with longer operation time, lower preoperative hemoglobin, ASA Physical Status > 2, total hip arthroplasty, anemia, autotransfusion, lower preoperative fibrinogen, and lower preoperative albumin were significantly associated with increased transfusion risk.

## Discussion

To optimize the utilization of blood products, improve outcomes and reduce healthcare costs, it is necessary to highlight the importance of preoperative evaluation and prediction for perioperative blood transfusion. Recent advancements in artificial intelligence are changing perioperative medicine for risk stratification, intraoperative monitoring, and intensive care management [32]. Machine learning has been regarded as a useful tool to process enormous data and accelerate the development of clinical prediction. To our knowledge, it is the first study to develop and test machine learning models to predict perioperative blood transfusion in hip surgery.

Although the calibration performances of models were below expectation, most algorithms showed good discrimination (AUC > 0.8) [29]. The Ridge Classifier had the best comprehensive performance and provided good net benefit for predicting perioperative blood transfusion. Unlike most previous studies, we focused on perioperative blood transfusion rather than intraoperative or postoperative transfusion, which decreased complexity and inconvenience of routine practice [33, 34]. Moreover, existing prediction tools have typically specialized in modeling a limited subset of surgeries or population and did not incorporate diagnoses to develop models [16, 26]. Our goal was to provide prediction models with generalizable application across a variety of procedures and diagnoses in hip surgery. Therefore, the performance might be not good as models for a single set of surgeries. Additionally, the model performances were assessed and compared by discrimination and calibration, accompanying with the accuracy, recall, precision, and F1 score of models, which greatly improved the reliability of results. Furthermore, although some studies have identified the risk factors of blood transfusion, the contribution of features to the transfusion risk was not characterized [16, 35]. This study extended interpretable visualization techniques to assist practitioners in early identification of important factors and potential interventions.

The variables utilized in the models were comprehensive and accessible in the hospital database. We combined patient demographic characteristics, preoperative laboratory tests, and surgical information to predict the likelihood of perioperative blood transfusion. Compared to previous studies, we included a large number of surgery-related variables to explore the association between them and the risk of perioperative blood transfusion. Consistent with previous studies, the machine learning algorithms outperformed logistic regression in our study and identify more risk factors [16, 36]. Machine learning models could extract information from large amounts of data and multiple variables, capture complex no-linear relationships and provide new hypotheses for clinical diagnosis and treatment. By exploring these relevant risk factors for perioperative blood transfusion in hip surgery, our findings could assist surgeons to identify high-risk patients and contribute to decision-making, including preoperative optimization, intraoperative monitoring tools, and postoperative management.

One of the strengths of our study was the visualization of machine learning model interpretation. Many machine learning algorithms produce models without providing the correlation between variables and outcomes. Understanding how machine learning models make predictions contributes to overcoming the drawback of the “black box” models and improving trust in machine learning for physicians [37, 38]. The risk factors screened by the Ridge classifier were consistent with clinical practice and previous literature. In agreement with previous studies, lower preoperative hemoglobin and higher ASA Physical Status have been regarded as important risk factors for transfusion in this study [16, 39, 40]. Additionally, anemia was a strong factor that increased the risk of transfusion. The prevalence of anemia in our cohort could reach 14.2%. Studies have shown that preoperative anemia was related to increased risk of blood transfusion [14, 41]. Evidence from a previous study demonstrated that the treatment of preoperative anemia was associated with decreased perioperative blood transfusion in patients undergoing elective orthopedic and gynecologic surgery [42]. Lower preoperative albumin has been identified as a risk factor for blood transfusion [43, 44]. The serum albumin level was a widely used marker of malnutrition, which has been demonstrated to be associated with postoperative complications and mortality following orthopedic procedures [45, 46]. Patients with lower albumin were more likely to be frail and in poor health status, which may increase transfusion requirements. As modifiable preoperative risk factors, patients may benefit from preoperative interventions to correct anemia and lower albumin level to avoid unnecessary blood transfusion. Preoperative fibrinogen was an important indicator of coagulation function. We found that lower preoperative fibrinogen was associated with increased transfusion risk, which has been examined in spine surgery [47].

As for surgery-specific variables, it was well-accepted that patients with longer operation time were at significantly increased risk of blood transfusion. Our study was consistent with past findings [16, 39]. Moreover, our data showed that the correlation between diagnosis and blood transfusion risk was important, which has been reported in previous studies. A study suggested that hidden blood loss after total hip arthroplasty significantly differed in patients with different diagnoses [48]. They found that blood loss in patients with nonunion of femoral neck fracture was increased in comparison with patients with osteoarthritis, avascular necrosis of the femoral head, and developmental dysplasia of the hip. Evidence from a previous study showed that patients who had ankylosing spondylitis with total bony ankylosis of the hips suffered more blood loss and blood transfusion than patients with hip osteoarthritis [49]. Another study reported that patients with rheumatoid arthritis appeared to have a significantly higher incidence of preoperative anemia and blood transfusion than osteoarthritis patients [50]. However, the types of ankylosing spondylitis and femoral neck fracture were not distinguished in our study. A possible reason for patients with femoral neck necrosis and osteoarthritis having the low transfusion rate may be because of the low difficulty and short duration of surgical procedures. Additionally, patients with osteoarthritis and femoral head necrosis were relatively common in our cohort. Surgeons were more experienced in surgical procedures, which may lead to less intraoperative blood loss. This may be another underlying reason for the results. Furthermore, it was necessary to note that machine learning algorithms could provide the correlation between variables and outcomes, but could not prove the causality. More investigations in large populations are needed to explore the relationship between diagnosis and blood transfusion risk. In terms of surgery types, total hip arthroplasty was regarded as a significant risk factor for blood transfusion. As a common and effective way for patients with hip disease to relieve pain and improve function, the demand for total hip arthroplasty has been rising in recent years [1]. Consistent with previous findings, the data from our study showed that patients with total hip arthroplasty were at the high risk of perioperative blood transfusion [3, 4]. These surgery-related risk factors should be also considered when preparing preoperative plans. Although some of them were not modifiable, effective interventions and strategies should be taken to reduce perioperative blood transfusion and avoid unnecessary health costs.

Recently, some attempts have been adopted to reduce blood transfusion in orthopedic surgery, such as the use of tranexamic acid and autotransfusion. Although several studies demonstrated the association between the use of tranexamic acid and decreased blood transfusion in hip arthroplasty, we did not find any significant difference in our cohort of hip surgery [51, 52]. The reason for this may be that tranexamic acid has been widely used in our institution and the population not using tranexamic acid was too small to detect the difference in the transfusion rates. Previous findings suggested that intraoperative autotransfusion was associated with reduced blood loss and transfusion [53, 54]. However, autotransfusion has been regarded as a risk factor for blood transfusion in our cohort, which may be attributed to the limited samples of autotransfusion in our cohort. Patients selected for autotransfusion were usually the ones who potentially would be at high risk of blood loss and they were more likely to receive perioperative blood transfusion. Although autotransfusion was applied in these patients, it could not decrease the need for allogeneic blood transfusion. For these patients, in addition to autotransfusion, other preoperative interventions should be taken to minimize allogenic blood transfusion.

There were several limitations in our study. First, this study was based on a single institution and lacked external validation. Second, there were some possible bias due to the retrospective nature of this study. Third, patients with missing data were eliminated, which may constrain the results of the analysis. Fourth, as for comorbidities, only hypertension, diabetes, and coronary artery disease were included, limiting further exploration of comorbidities on perioperative blood transfusion. Fifth, the period of data was relatively large and there may be some unknown time-related effects. Finally, it is necessary to acknowledge that the odds of transfusion could not be represented based on present analysis, which is the common limitation in the studies of prediction models [16, 33, 34].

## Conclusions

We developed and tested machine learning models with excellent discriminative ability to predict perioperative blood transfusion in hip surgery, which may allow surgeons to support decision-making, improve clinical outcomes and reduce healthcare costs. Identification of risk factors provides opportunities for accurate evaluations and prompt interventions. Further studies are still needed to validate the models in large cohorts and expand clinical implementation.

## Data Availability

The datasets generated and analyzed during the current study are available from the corresponding author on reasonable request.

## Abbreviations

RBC: Red blood cell
SHAP: SHapley Additive exPlanations
ASA: American Society of Anesthesiologists
BMI: Body mass index
APTT: Activated partial thromboplastin time
PT: Prothrombin time
ALT: Alanine aminotransferase
Min: Minutes
AUC: Area under the receiver operating characteristic curve
OR: Odds ratio
CI: Confidence interval
